# Psychological Capital and University Students’ Entrepreneurial Intention in China: Mediation Effect of Entrepreneurial Capitals

**DOI:** 10.3389/fpsyg.2019.02984

**Published:** 2020-01-23

**Authors:** Jianbo Zhao, Guojiang Wei, Kou-Hsiung Chen, Jui-Mei Yien

**Affiliations:** ^1^Institute of Industrial Economics, Chinese Academy of Social Sciences, Beijing, China; ^2^School of Economics, Fujian Normal University, Fuzhou, China; ^3^Department of Business Administration, Cheng Shiu University, Kaohsiung City, Taiwan; ^4^Department of Business Administration, University of Kang Ning, Tainan, Taiwan

**Keywords:** psychological capital, entrepreneurial intention, entrepreneurial capital, SEM, university students

## Abstract

The aim of this study was to identify the influences of psychological capital (PC) on students’ entrepreneurial intention (EI) in China’s universities. The mediating effects of Entrepreneurial Capitals were also examined. Based on the analysis of the traditional capital and PC, the paper proposes that traditional capital is the direct factor to drive the behavior of entrepreneurship, while psychological factors do not directly affect EI, but improve EI by influencing traditional capital. A total of 1914 responses from universities in southeast China were analyzed using the structural equation modeling (SEM) approach to test study hypotheses. Results show that PC has a significant indirect impact on students’ EI only through traditional financial, human, and social capital (SC). These results support the mediating role of the traditional entrepreneurial capitals in explaining the relationship between PC and EI. Additionally, the impact of SC on EI is higher than that of financial and human capital (HC). Finally, research limitations and implications are discussed and future research directions are suggested.

## Introduction

Entrepreneurship is an important way to improve a nation’s economy (Kadiyono and Ashriyana, 2017), and it can provide more employment opportunities and accelerate innovation. Entrepreneurship can also solve problems such as social and environment challenges by setting up new firms and the application of new technology and products (Stephan et al., 2016). The success of American college students’ entrepreneurship and Silicon Valley has made many countries realize the importance of college students’ entrepreneurship. Most countries in the world have adopted various means to accelerate entrepreneurship, especially university students’ entrepreneurship, which can accelerate the application of new technologies and promote innovation. The rising unemployment rate after the 2008 financial crisis has further prompted local governments to take steps to promote university students’ entrepreneurship (Saputri, 2016). However, the earliest research on entrepreneurship was from the perspective of economics, and the researches explained entrepreneurial activities with standard economic models, and assuming perfect rational economic man (Obschonka, 2017). Economics analyze entrepreneurship from the general and macro characteristics and draw the uniform conclusion. Researches based on economic perspective neglect entrepreneurs’ heterogeneity and can’t reveal the difference of individual entrepreneurial process. Each entrepreneur has different backgrounds, objectives, and aspirations. Some of them are the innovators who reject society’s prevailing norm and want to live a freestyle life (Teal and Carroll, 1999). Some entrepreneurs are forced to earn a living and some want to realize their dream by setting up new firms. They are special in their personality and entrepreneurship can’t be explored only by a simple hypothesis of rational economic man. Entrepreneurship must be analyzed from the perspective of individual context, especially considering its differences in background and the entrepreneurial capitals they occupied.

Scholars have studied the impact of traditional capital and psychological capital (PC) on the performance at workplace and enterprises, but they mostly took the capitals as juxtaposition factors, and have not analyzed their impact on entrepreneurial intention (EI) or willingness. This paper explores the impact of university students’ PC on traditional entrepreneurial capital and ultimately the impact on EI. The study is presented as follows: Section “Theoretical Background and Hypotheses” explains the theoretical background and proposes the hypothesis. Section “Materials and Methods” outlines the details of the data and research method. Then, Section “Empirical Results” gives the results, and finally, Section “Conclusion and Implications” elaborates the conclusion, implications, limitations, and future research.

### Theoretical Background and Hypotheses

In economics, factors of production are crucial for competition and business success, which means financial and human factors are very important in entrepreneurship. Later, social capital (SC) (Schumpeter, 1909; Metzler, 1951) was introduced in the analysis and they are often referred to as traditional capital. There are many studies on the impact of traditional capital on entrepreneurship (Cooper et al., 1994; Honig, 1998; Kim et al., 2006; Orser et al., 2006; Cetindamar et al., 2012), but no research analyzes what influences the traditional capital. In the 21st century, scholars introduced PC and symbolic capital (Shaw et al., 2009) to analyze entrepreneurship. Many scholars combine PC with traditional capital to study performance at work. Although there have been many studies on PC in the past two decades, most of them took PC as an independent factor, without analyzing the relationship between financial, human, SC, and PC and their impact on college students’ EI. Furthermore, the research regards financial, human, SC, and PC as parallel elements, and fails to analyze their inter-correlation and working mechanism. This paper suggests that different capital plays a different role in entrepreneurship and believes that there are some mechanisms among the four types of entrepreneurial capital, and analyze the relationship between them by structural equation modeling (SEM).

#### Traditional Entrepreneurial Capital

Entrepreneurial capital is a concept that has been widely concerned since the rise of entrepreneurship, and its extension has been expanding with time (Firkin, 2001; Erikson, 2002; Vinturella and Erickson, 2003; Fletschner and Carter, 2008). In general, entrepreneurial capital includes economic, human, social, psychological, and symbolic capital (Shaw et al., 2009). Among them, economic (or financial), human, and SC are often referred to as traditional capital.

From the perspective of economics and managerial practice, starting up a business needs financial capital (FC) and human capital (HC). Financial and HC needed for setting up business will positively influence the success of entrepreneurship. FC includes both financial and tangible assets such as plant and equipment. In the early days of industrial revolution, technological progress was relatively slow, and FC was an important condition for the development of enterprises. FC is the prerequisite to set up enterprises. The stock of FC to support a new venture and its growth has been proven a key factor (Nasurdin et al., 2012). Therefore, this paper assumes that individual FC has a positive role in promoting EI.

Classical economics holds that the main factor of production of firms is FC, while labor force is subordinate to FC. With the increase of the amount of FC and the intensification of competition among enterprises, the role of pure FC in entrepreneurship begins to decline. Alfred Marshall emphasized the importance of HC (Marshell, 1890), Schultz clearly pointed out that HC is the main reason for promoting national economic growth in the present era (Theodore, 1990). With the increasing recognition of new resources as a competitive advantage in global competition, HC and SC are important for enterprises and being touted completely.

Human capital refers to the knowledge, skills, abilities, or competencies derived from education, experience and specific identifiable skills (Luthans and Youssef, 2004). The essence of HC is the necessary knowledge system to start a new venture. With the development of industrial revolution, technology has become an important recognition of enterprise competition, and the role of HC has become increasingly prominent, while the importance of FC has declined due to the development of financial derivatives. The condition of enterprise development is the guarantee of entrepreneurship, and HC in entrepreneurship is becoming important. Martin’s empirical research reveals that there is a significant relationship between entrepreneurship-related HC assets and entrepreneurship outcomes (Martin et al., 2013). Murat found that the entrepreneurial HC plays a relatively more important role (Iyigun and Owen, 1998). The article believes that HC has a positive effect on entrepreneurship.

Social capital refers to the resources you can turn to for help both inside and outside a firm (Luthans et al., 2004). SC is an extension to financial and HC, which involves the size, structure, and composition of networks (Portes, 1998). SC means the ability of actors to secure benefits by virtue of membership in social networks or other social structures, which can bring better performance of the organization. In the process of economic transformation from shortage to surplus, the focus of business management concepts has shifted from production to products and to marketing and social marketing. SC has become a new form of capital beyond financial and HC. People with broad extensive SC, namely, internal and external networks, can turn to more resources to solve the business problems. Adler and Kwon (2002) found that SC has a positive impact on both HR and organizational areas (Adler and Kwon, 2002). Many works proved that SC has a positive relationship with entrepreneurship (Cope et al., 2007; Light and Dana, 2013). Batjargal found that the interaction of SC and experience of entrepreneurs (human capital) has a positive effect on the survival likelihood of firms (Batjargal, 2007). SC can provide entrepreneurs with information, market, and other resources to ensure the success of entrepreneurship. The paper proposes that SC is also crucial to entrepreneurship. Based on the research above, the paper proposes the following hypotheses.

*Hypothesis 1a.* Financial capital influences the entrepreneurial intention positively.
*Hypothesis 1b.* Human capital influences the entrepreneurial intention positively.
*Hypothesis 1c.* Social capital influences the entrepreneurial intention positively.

#### Psychology Capital

Many studies have confirmed that financial, human, and SC have an important positive relationship with job performance, satisfaction, and innovation. Some researches analyze traditional capital with newly emerging psychology capital together. Larson and Luthans empirically studied that human, social, and PC have positive effects on organizational loyalty and job satisfaction, and the correlation between PC and job satisfaction is higher than that between HC and SC, and the correlation between PC and organization is also much higher than that of SC (Larson and Luthans, 2006). Xiong explores the impact of human, social, and PC on innovation performance and finds that the human, social, and PC of the new generation of knowledge workers have significant positive effects on job satisfaction and innovation performance, respectively (Xiong et al., 2018). Ke’s empirical study finds that HC, SC, and PC have positive correlations on entrepreneurship. They all have significant positive effects on task performance and contextual performance, but PC has the strongest influence, SC takes the second place, and HC is the weakest (Ke et al., 2010).

However, the researches above analyze the impact of financial, human, and SC on entrepreneurship together. This paper argues that the impact of four capitals on entrepreneurship is not at the same level, some of which have direct and some have indirect effects.

Psychological capital (PsyCap in short) initially was only used in the economics literature to study its relationship to wages (Goldsmith et al., 1998; Kossek et al., 2003) and deviates from positive psychology in the late 1990s, which emerged with a renewed emphasis on what is right with people, instead of what is wrong with people (Seligman and Csikszentmihalyi, 2000; Snyder and Lopez, 2002). Martin Seligman introduced the positive psychology and emphasized the important function of positive psychology (Seligman, 1975; Abramson et al., 1978). The positive psychology has two developments: the macro-oriented positive organizational scholarship or POS movement (Cameron et al., 2003) and the micro-oriented, state-like positive organizational behavior or POB approach by Luthans. Luthans defines PsyCap as an individual’s positive psychological state of development, including self-efficacy, optimism, hope, and resiliency (Luthans, 2002). The POB theory proposed to attaching importance to the management and cultivation of psychological factors (Thomas and Tankha, 2018). Luthans puts forward the difference between positive PC and HC and SC and the way of management and development (Luthans and Youssef, 2004; Luthans et al., 2004). Luthans believes the unique feature of PC is that it is “state-like” and open to development. Luthans pointed out that POB emphasizes positively oriented human resource strengths named psychological capacities. His works reveals the feature of PsyCap and concludes that it can be measured, developed, and effectively managed for performance in today’s workplace (Goldsmith et al., 1998). He developed a highly focused, 2-h web-based training intervention and concluded that PC can be developed (Luthans et al., 2008b).

Psychological capital includes many aspects. Jiang and Na (2013) believe that PsyCap includes six facets of self-efficacy, hope, optimism, resiliency, opportunity recognition, and social ability. Ke’s research indicates that the construct of PC has two factors: task-oriented PC including self-confidence and courage, optimism and hope, spirit of enterprise and diligence, resiliency and perseverance, and relation-oriented PC including toleration and forgiveness, respecting and courtesy, modesty and prudence, thanksgiving and dedication (Ke et al., 2009). Bockorny and Youssef-Morgan (2019) proposed that courage is also valuable as hope and optimism, and entrepreneurs’ courage is related to their life satisfaction and the venture. At present, the popular classification is determined by Luthans in 2006 and they are self-efficacy, hope, optimism, and resiliency.

Psychological capital affects one’s career and personal achievement and influences people’s behavior in many ways. PC emphasizes more on power, success, embellishment, and happiness (Donaldson, 2013), and it can promote performance and increase satisfaction.

Generally, the PC has impacts on all workplace attitudes and performance, including employees’ satisfaction, employees’ presentation (Avey et al., 2010; Larson et al., 2013). Many empirical researches illustrated that an employee with higher PC gives better performance at the workplace than an employee with lower PC (Reynolds et al., 2005). In 1998, Luthans conducted a meta-analysis of self-efficacy and confirmed the correlation between self-efficacy and work-related performance (Stajkovic and Luthans, 1998). Luthans’ study indicates a significant positive relationship regarding the composite of the four facets with performance and satisfaction (Kossek et al., 2003). In 2007, Luthans put forward the measurement tools and methods of PC and analyzed the relationship between job performance and job satisfaction through empirical research (Luthans, 2002).

Psychological capital also affects people’s entrepreneurship. The meta-analytic findings showed that PsyCap such as self-efficacy and need for achievement, and entrepreneurial orientation are highly associated with entrepreneurship (Frese and Gielnik, 2014) and also reveal a small, positive relationship between personality and risk-taking propensity and new venture creation and success, and a moderate, positive relationship between innovativeness, need for achievement, and self-efficacy and new venture creation and success. Some researchers also reveal that EI is related to all dimensions of PC, especially with self-efficacy and resilience. PC as an integrated construct is related to EI as a whole (Contreras et al., 2017). In other words, the entrepreneurs are high in PC. Hmieleski found that entrepreneurs’ PC could explain significantly variance in new venture performance, above and beyond measures of FC, HC, and SC (Hmieleski and Carr, 2008).

The researchers believe that PC can influence the entrepreneurship directly. But we hold that PC could not be seen as a factor of production and can’t determine the result of entrepreneurship. PsyCap can affect the entrepreneurs’ ability to acquire the financial, human, and SC and there must be a certain correlation between entrepreneurial capitals. From the literature, we find that there are more achievements in the study of PC alone, but less work on entrepreneurship analyzing human, social, and PC together, and let alone FC. The process of entrepreneurship is an economic activity, so the factors of production in economics are crucial for setting up business. Martin’s research found that optimism significantly moderates the relationship between entrepreneurial capitals and the success of new business. Both entrepreneurial capitals and PC are significant predictors of entrepreneurial success (Martin et al., 2016). Traditional capital plays a direct role in the process of entrepreneurship, while PC does not directly affect entrepreneurship. So, the paper proposes that the PsyCap doesn’t influence the entrepreneurship directly but exert impact on the factors to startup business such as financial and HC. The empirical study showed that the employees’ PsyCap had a significant additional impact over human and SC on these work attitudes and job satisfaction (Iyigun and Owen, 1998). The Attraction-Selection-Attrition (ASA) theory suggests that PC is negatively related to stress (Baron et al., 2016). Therefore, this paper proposes that the PC determined by personality will affect the individual’s ability to acquire traditional capital and proposes the following hypotheses.

*Hypothesis 2a.* Psychological capital affects the financial capital positively.
*Hypothesis 2b.* Psychological capital affects the human capital positively.
*Hypothesis 2c.* Psychological capital affects the social capital positively.

#### Mediating Roles of Traditional Entrepreneurial Capitals on Psychological Capital and Entrepreneurial Intention

For most entrepreneurs, PC is the important factor determining the entrepreneurship. Most entrepreneurs regularly confront the shortages of financial, human, and SC, and they have only themselves and their psychological state to rely on to get the job done (Hmieleski and Carr, 2008). The PC, especially hope and self-efficacy can promote entrepreneurial passion, which is a driver and source of energy to work hard and persist in the process (Baum and Locke, 2004). Optimism and resilience can make entrepreneurs believe in the feasibility and success of an idea in entrepreneurship characterized by enormous uncertainty and a high degree of complexity (Baum and Locke, 2004) PC can produce strong entrepreneurial motivation, which positively affects the entrepreneurial decision-making process (Baron et al., 2016).

As discussed before, only the intention is not enough for innovation and entrepreneurship. Especially, graduates may leave universities or colleges with academic knowledge but it is not enough to help them tackle the problem of entrepreneurship because they are totally disconnected from their social environment. SC contributes an overtly social and interpersonal element to entrepreneurial behaviors, with access to entrepreneurial opportunities. Similarly, entrepreneurial ability to realize opportunities in the marketplace is greatly influenced by HC. Considering the relative scarcity of capital in earlier period of entrepreneurship, FC is the most important resource for setting up business. Therefore, the entrepreneurial capitals of human, financial, and social will enhance the relationship between the psychological intention and entrepreneurial behaviors, which connect universities and industry, and enhance the student experiences, equipping the graduates with capitals of knowledge and skills in their entrepreneurial areas. We propose the following hypotheses.

*Hypothesis 3a.* Social capital mediates the relationship between psychological capital and entrepreneurial intention.
*Hypothesis 3b.* Human capital mediates the relationship between psychological capital and entrepreneurial intention.
*Hypothesis 3c.* Financial capital mediates the relationship between psychological capital and entrepreneurial intention.

But as the basic elements of entrepreneurship capitals, their contributions to a successful upstart might be different. The importance of FC is decreasing currently due to affluent financing opportunities and various financing channels. The HC of the enterprise, that is, the knowledge of the market and the technology, creating a network of resources around them that can be used for entrepreneurship, has become a key factor for the success of business, so the mediation effect of PC on HC is greater than that of FC. Simultaneously, because of the importance of cooperation in a market economy, the mediation effect of SC is also higher than that of economic capital. So we put forward the following hypotheses.

*Hypothesis 4a.* The mediation effect of human capital on psychological capital and entrepreneurial intention is greater than that of FC.
*Hypothesis 4b.* The mediation effect of social capital on psychological capital and entrepreneurial intention is greater than that of financial capital.

**FIGURE 1:**
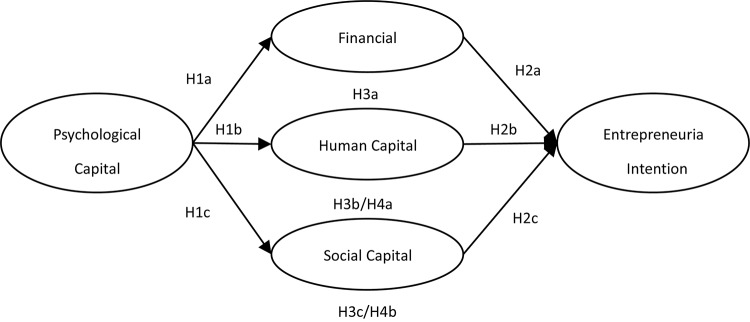
The hypothesized model.

Figure 1 shows the hypothesized model depicting the relationships among PC, traditional entrepreneurial capitals, and their effect on EI.

## Materials and Methods

### Measurement

The purpose of this study was to explore the relationships between PC (PsyCap or PC in short), FC, HC, SC, and EI in a sample group of students in the universities of Southeast China. The following hypotheses were tested: (1) PC is positively correlated with the level of FC, HC, and SC. (2) FC, HC, and SC have positive effects on EI. (3) FC, HC, and SC partially mediate the relationship between PC and EI.

To test our hypotheses, the study conducted a questionnaire survey. The questionnaire consists of three parts, entrepreneurial capital including PsyCap, financial, human, and SC (see Table 1), EI, and sample information.

**TABLE 1:** The structure of the questionnaire.

Contents			Items
Entrepreneurial capital	PsyCap	Self-efficacy	4
		Hope	4
		Optimism	4
		Resilience	4
	Social capital		3
	Human capital		3
	Financial capital		3
Entrepreneurial intention			5
Sample information			4

The earliest measurement of PC was carried out separately on the four elements of PC, among which Snyder measured hope (Snyder et al., 1996), Wagnild and Young measured resilience (Wagnild and Young, 1993), Scheier and Carver measured optimism (Scheier and Carver, 1985), and Parker measured self-efficacy (Parker, 1998).

Then, PsyCap was measured with the 24-item PsyCap Questionnaire (PCQ) (Luthans et al., 2015; Luthans, 2002) by Luthans et al. (2008a) with six items for each of the four components (efficacy, hope, resilience, and optimism), PCQ demonstrated adequate confirmatory factor analytic structure across multiple samples and had strong internal reliability (α = 0.88). In order to reduce the fatigue and boredom of the respondents, a short version of the scale with 16 items was used in this study to measure the PsyCap. We selected four of six 24-item PCQ and adapted it a little so as to be suited for Chinese students. Because most college students have no experience in the workplace, we modified the related items into items about their study and lives. To get a composite PsyCap score, all four responses for each of the four subscales were summed and averaged to get a subscale composite average.

Financial capital mainly refers to the acquisition of or various external financing, technology, and equipment that are needed for setting up new business. The questionnaire designs three items to survey the financial status of college students, including families’ financial support for entrepreneurship, their own financial resources and technology, and equipment obtained through external channels.

Human capital can be expressed by education and working years (Snell, 1999), but college students do not have working experience, so it is expressed by three items: students’ experience, knowledge and skills, and entrepreneurial ability.

Leana’s Social Capital Scale is mainly expressed in terms of the number of participating organizations or activities (Leana and Van Buren, 1999). However, this study considers that SC is mainly the entrepreneurial support that individuals can obtain from outside, so it mainly includes three items: the status of support provided by social relations for entrepreneurship, the role of learning environment for entrepreneurship and the support of family and relatives and friends for entrepreneurship.

Entrepreneurial intention is based on Thompson’s individual entrepreneurial intent Scale (IEIS), and we select five items related to students’ entrepreneurship. The items that are reverse coded in scale are changed into obverse ones.

### Sample Collection and Data

We used previously published and validated measures in this study. The questionnaires comprised demographic questions (e.g., age, sex, major and educational level) and five variables. The independent variables were the PC, FC, HC, and SC. The EI with five items served as dependent variables in the model. All questions were rated on a five-point Likert scale (strongly disagree = 1, strongly agree = 5). Reliability testing of the scales was conducted before the questionnaires are distributed.

Survey data were collected from a random sample of university students in Southeast China, because Southeast China is well-developed and people have traditional business spirit, has a good market environment, and is currently the region with the most active entrepreneurial and innovative activities. We received a total of 2039 completed questionnaires with a return rate of 82%, among which 1914 are qualified. Table 2 shows means, standard deviations, and correlations of the study variables. Scale reliability was tested by calculating items for total correlation coefficients and Cronbach’s alpha for the overall scale. The composite reliability of each of the constructs was greater than 0.7, which indicates that all the variables meet the requirement of construct reliability.

**TABLE 2:** Descriptive statistics and variable inter-correlation.

	Mean	SD	Inter-correlation							
			Self-efficacy	Hope	Resilience	Optimism	PsyCap	Financial capital	Human capital	Social capital
Self-efficacy	3.699	0.743								
Hope	3.533	0.723	0.752							
Resilience	3.539	0.773	0.695	0.746						
Optimism	3.508	0.780	0.594	0.681	0.702					
PsyCap	3.570	0.660	0.867	0.901	0.895	0.859				
Financial capital	3.033	0.905	0.562	0.616	0.620	0.583	0.677			
Human capital	2.895	0.950	0.559	0.628	0.604	0.589	0.677	0.775		
Social capital	2.944	0.941	0.530	0.630	0.624	0.602	0.679	0.791	0.863	
Entrepreneurial intention	2.691	1.031	0.517	0.552	0.541	0.481	0.595	0.674	0.730	0.719

**N* = 1914.*

The PsyCap of college students is quite high, and each of the facets is higher than the other traditional capital. The students’ HC is the lowest among the four types of capital, and their EI is very low. From the perspective of different individuals’ context, the scores of boys are higher than those of girls, and the scores of the students majored in science and engineering are higher than those of the students majored in liberal arts. The statistics demonstrate that boys prefer entrepreneurship more and the students majored in science and engineering have the advantage in technology.

## Empirical Results

The goodness of fit of the models was evaluated using absolute and relative indices. The absolute goodness-of-fit indices calculated were the chi-square goodness-of-fit statistic, RMSEA, GFI, NNFI, IFI, and CFI. Non-significant values of chi-square indicate that the hypothesized model fits the data. Values of RMSEA smaller than 0.08 indicate an acceptable fit and values greater than 0.1 means the model should be rejected. Relative-fit index values are greater than 0.90, indicating a good fit.

Given the multivariate nature of the variables in the model and the need to assess both the measurement properties of the scales and the substantive relationships between them simultaneously, SEM (AMOS) with maximum likelihood estimation was used. In the paper, we take the FC, HC, and SC as mediators, and conduct Mediation Effect analysis. When considering the influence of independent variant PC on dependent variant EI, the influences are not directly but through these mediators. Therefore, AMOS was used to test two models: the one is a basic or direct model (PC→EI); the other is a fully mediated model that includes mediation effects (PC→FC→EI; PC→HC→EI; PC→SC→EI).

We discuss the CFA analysis and measurement model of PC firstly, followed by the substantive model. We first tested the measurement model of PC to assess the items’ correspondence to their respective latent variables. Briefly, parceling combines items randomly into following a parceling procedure for our constructs, and scales’ items were parceled randomly into composites indicators, which entered the measurement model as multiple indicators to estimate their respective latent variables. The measurement model resulted in excellent fit statistics. Specifically, NFI (0.95), RFI (0.94), IFI (0.96), TLI (0.95), and CFI (0.95) exceeded the recommended 0.90 cutoff, and RMSEA (0.06) was below the recommended 0.10 level. In combination, these suggest uni-dimensionality of the scales used. The CFA model’s chi-square was significant (chi-square = 893.64 with 100 degrees of freedom; *p* = 0.0001). So, the test of reliability of PC scales in the survey is acceptable.

**FIGURE 2:**
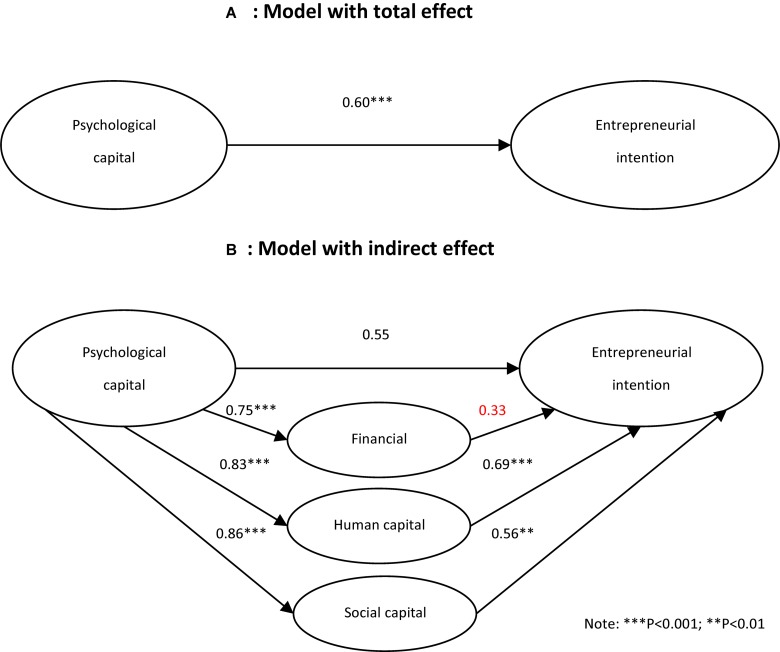
Path model analysis output.

Exploring the mechanism of PC on EI, we use SEM. Raw data were used for the path analysis, and Table 3 shows the results of model analysis. According to Figure 2, in the basic model, there is a direct and positive relationship between PC and EI. This is not surprising given the large sample size. The model fit statistics were excellent and within acceptable cutoff rates. RMSEA (0.06) was lower than the traditional 0.10 cutoff level. NFI (0.94), RFI (0.94), IFI (0.95), TLI (0.94), and CFI (0.95) were all higher than the 0.90 cutoff advocated by Hair et al. (2014). The model’s chi-square was significant (chi-square = 1651.84 with 184 degrees of freedom; *p* = 0.0001). The result of the basic model shows that PC is an important factor influencing students’ EI and the total impact is 0.60.

**TABLE 3:** Structural model results.

	Model 1 (Basic model)				Model 2 (Full model)			
Relationship	Estimate	S.E.	C.R.	*P*	Estimate	S.E.	C.R.	*P*
PC→FC					0.751	0.023	32.443	***
PC→HC					0.829	0.019	43.729	***
PC→SC					0.859	0.020	43.956	***
FC→EI					0.328	0.080	4.087	0.160
HC→EI					0.691	0.077	8.973	***
SC→EI					0.564	0.075	7.507	**
PC→Self-efficacy	0.625	0.018	33.870	***	0.524	0.018	28.424	***
PC→Hope	0.667	0.018	37.308	***	0.585	0.018	32.878	***
PC→Resilience	0.669	0.018	37.263	***	0.589	0.018	32.800	***
PC→Optimism	0.546	0.021	26.246	***	0.519	0.020	25.371	***
PC→EI	0.595	0.037	15.989	***	0.551	0.148	3.729	0.526

***P < 0.001; **P < 0.01.

In the full model, when we introduce Financial Capital (FC), Human Capital (HC) and Social Capital (SC) as mediators in the relationship between PC and EI. The test results shows that the relationships between psychological capital (PC) and three mediators, and three mediators and EI, are significant except FC and EI. The results demonstrate psychological capital has impact on financial capital (*r* = 0.75, *p* < 0.001), human capital (*r* = 0.83, *p* < 0.001), social capital (*r* = 0.86, *p* < 0.001), which verifies the hypothesis of H1a, H1b, H1c. Simultaneously financial, human, social capital influence university students’ entrepreneurial intention directly, and the effect of HC on EI (*r* = 0.69, *p* < 0.001), SC on EI (*r* = 0.56, *p* < 0.01) is significant, FC on EI (*r* = 0.33, *p* > 0.05) is not significant, which testing the hypothesis of H2b and H2c, but H2a is not supported. The conclusion is also consistent with the theory of the convertible nature of entrepreneurial capitals by Shaw.

The testing for mediation to determine how the intervening variable (mediator) transmits the effect of an independent variable to a dependent variable was performed. To avoid Type I and Type II errors in testing our mediating effect, joint significance of the two effects comprising the intervening variable effect and independent variable was adopted as stipulated by MacKinnon et al. (2002).

It can be seen from Figure 2 that the coefficient for PC on EI dropped significantly from 0.60 to 0.55 when SC, HC, and FC are introduced as mediators. After introducing three mediators, the relationships between PC and EI is not significant (*p* = 0.526), which is 0.595 (*p* < 0.001) before. The results suggest that SC, HC, and FC may be exerting a mediation effect. The findings support H3a, H3b, and H3c, suggesting that perceptions of PC indirectly predict EI through the mediating role of FC, HC, and SC. So, we could say that FC, HC, and SC mediate the relation between PC and EI. The measurement model resulted in NFI (0.90), RFI (0.90), IFI (0.91), TLI (0.90), and CFI (0.90), and all exceeded the recommended 0.90 cutoff, and RMSEA (0.05) was below the recommended 0.10 level. The model’s chi-square was significant (chi-square = 4076.19 with 394 degrees of freedom; *p* = 0.0001). Given the fit statistics, we proceeded to examine the estimated coefficients (Table 3). Therefore, from mediation analysis, it is confirmed that SC, HC, and FC mediate the relationship between PC and EI.

Regarding the degree of contributions, the results show that the mediation effect of PC on the financial, human, and SC is 0.248, 0.573, and 0.482, respectively, demonstrating that H4a and H4b are correct. China’s entrepreneurship environment has shifted to high-level economic development based on knowledge and technology, and the role of FC in entrepreneurship and economic development is declining.

## Conclusion and Implications

The paper investigates the relationship between PC and EI, as mediated by FC, HC, and SC. The paper reveals that PC has a mediating effect on college students’ EI in China by influencing social, human, and economic capital, which supports hypothesis 3 of this study. All the results support the proposed hypotheses. This research could enhance knowledge of these factors, and the results could be expanded in future experimental studies.

### Main Conclusion

This paper clearly reveals the relationship between various forms of entrepreneurial capital, and proves that the PC is positively related to FC, HC, and SC, and explains the mechanism how PC enhances entrepreneurial willingness. The research emphasizes the role of PC in promoting entrepreneurship, linking economics with management.

(1) The effect of PC on entrepreneurship intention is not direct, but through FC, HC, and SC to influence students’ EI.
(2) The four facets of PC play an unequal role in the construct of PC. The coefficients of self-efficacy, hope, resilience, and optimism are 0.52, 0.59, 0.59, and 0.52, respectively. Self-efficacy is the most important component of PC. This is nearly the same as Rauch’s conclusion that self-efficacy has a stronger association with success especially (Rauch and Frese, 2007). Many researches reveal that self-efficacy has a positive impact on work-related performance (Bandura, 1997). Harudin also proved that the strongest relative influence toward the EI is self-efficacy (Harudin et al., 2016).
(3) When setting up a new business, the role of traditional financial, human, and SC contributing to the behaviors of entrepreneurship is different, which is 0.33, 0.69, and 0.56, respectively. In traditional entrepreneurial capitals, the role of FC in promoting entrepreneurship is much lower than that of HC and SC. With the maturing financial market such as venture capital, the degree of its contribution to entrepreneurship is declining. The significant impact of HC and SC on entrepreneurship intention shows that China’s economic development has shifted from capital-driven to human capital-driven and social capital-driven, and the contribution of technology and social network to economic development is constantly emerging.
(4) Psychological capital plays an important role in increasing FC, HC, and SC, which is rarely involved in the literature. Some researchers have paid attention to the relationship of traditional capital. For example, Danes summarizes the influence of family capital (human, social, and financial) on financial success of family firms (Danes et al., 2009). Luthans noted that SC contributes to the creation of HC (Luthans and Youssef, 2004). But no research studies the impact of PC on traditional capital.
  The influence coefficients of PC on financial, human, and SC are 0.75, 0.83, and 0.86, respectively. Psychological capital has the weakest impact on FC and the most significant impact on SC, which fully proves the important role of PC in social and economic development. PC has become an important form of modern capital. PC can not only improve job performance, job satisfaction, and innovation (Donaldson, 2013), but also enable individuals to overcome difficulties in order to obtain more financial support and improve financing capacity and FC. PC can enable individuals to have higher self-efficacy, so as to formulate more scientific and reasonable individual learning plans, improve self-control and perseverance to acquire more knowledge to set up business. The psychological factors were strongly associated with SC outcomes (Steinfield et al., 2008). PC can enable individuals to get along with colleagues and classmates more optimistically, form a larger network of social organizations, and have more SC.

(5) At present, the most important factor affecting university students’ EI in China is the insufficient traditional capital, which has become an important factor affecting students’ entrepreneurial willingness.

### Managerial Implications

Increasing entrepreneurship spirit in university study is a key factor affecting the university students’ EI. Actually, only the PC is not enough to improve the EI. Therefore, this study established a mediation effect (hypothesis 3) in which social, human, and FC mediated the relationship between PCs on EI. While PC alone cannot lead to entrepreneurship, tests indicated that the level or the degree at which three capitals contribute to entrepreneurship is different, and the FC is weaker compared to social and HC. Hence, education is not only the source of HC, but also the source of entrepreneurial willingness and SC. Successful business management depends on both HC and SC (Belliveau et al., 1996; Podolny and Baron, 1997). The important foundation of human and SC is to improve students’ PC. The positive PC can be invested in and managed as human and SC, but with less monetary cost (Luthans et al., 2004). In the cultivation of university students’ entrepreneurship ability, we should focus on the cultivation of students’ PC. At present, there are fewer courses on the development of PC in university education, and less research and practice on the cultivation of university students’ PC.

The curriculum of entrepreneurship education should be diversified to provide more ways and skills for students to acquire startup capital. It is very important to give students more opportunities to participate in social organization to form more SC.

### Limitation and Future Research

Firstly, the sample size of this study is small, which can only explain the general situation of students’ entrepreneurial capital in Fujian Normal University, but cannot explain the overall situation of the students in the whole country. Secondly, the paper finds that there are differences in startup capital and willingness among students of different genders and majors, but there is no in-depth analysis.

The general situation of college students’ startup capital in China, the mechanism of PC affecting traditional capital, and the reasons for the difference of students’ PC can be researched in the future.

## Data Availability Statement

The datasets generated for this study are available on request to the corresponding author.

## Author Contributions

GW and JZ: conceptualization, methodology, and formal analysis. JZ and K-HC: software. JZ: writing – original draft preparation. GW and J-MY: writing – review and editing.

## Conflict of Interest

The authors declare that the research was conducted in the absence of any commercial or financial relationships that could be construed as a potential conflict of interest.
